# Ultrahigh-Q Polarization-Independent Terahertz Metamaterial Absorber Using Pattern-Free Graphene for Sensing Applications

**DOI:** 10.3390/nano14070605

**Published:** 2024-03-29

**Authors:** Youxin Chen, Guotao Sun, Jiang Wei, Yan Miao, Wenqian Zhang, Kaiyu Wu, Qingkang Wang

**Affiliations:** 1 National Key Laboratory of Advanced Micro and Nano Fabrication Technology, Shanghai Jiao Tong University, Shanghai 200240, China; usingchen@sjtu.edu.cn (Y.C.); sunguotao@sjtu.edu.cn (G.S.); wei_jiang86@sjtu.edu.cn (J.W.); miao_yan@sjtu.edu.cn (Y.M.); zwq0618@sjtu.edu.cn (W.Z.); 2 Department of Micro/Nano Electronics, School of Electronic Information and Electrical Engineering, Shanghai Jiao Tong University, Shanghai 200240, China

**Keywords:** graphene, terahertz metamaterial absorber, Q factor, refractive index sensing

## Abstract

In contrast to noble metals, graphene exhibits significantly lower loss, especially useful for optical sensing applications that require ultrahigh Q factors, and offer wide range tunability via an adjustable Fermi level. However, precise graphene patterning is difficult, especially for large areas, severely limiting its applications. Here, a tunable terahertz metamaterial absorber (TMMA) with ultrahigh Q factors consisting of a continuous, pattern-free graphene is demonstrated. A graphene sheet is overlaid on an Al metal array, forming a structure that supports strong localized surface plasmon polaritons (LSPPs) with fields tightly confined in the graphene, minimizing loss. Theoretical results show that this TMMA exhibits an ultrahigh Q factor of 1730, a frequency sensitivity of 2.84 THz/RIU, and an excellent figure of merit (FoM) of 365.85 RIU^−1^, independent of polarization. A tunability from ~2.25 to ~3.25 THz is also achieved by tuning *E_f_* of graphene from 0.3 to 0.7 eV. The proposed graphene-based TMMA holds many potential applications, particularly in the field of sensing.

## 1. Introduction

Metamaterial, an artificially engineered composite material, demonstrates extraordinary optical properties absent in natural materials [1,2,3]. It proves to be a promising candidate in various applications, including negative refraction, perfect lens, electromagnetic stealth, and absorbers [4,5,6,7]. A noteworthy application of metamaterials is the metamaterial absorber (MMA), extensively employed across electromagnetic bands from microwaves to optical frequencies [8]. Due to the limited interaction of natural materials with THz waves, metamaterial has opened up a new way to interact with THz waves. Recently, a variety of terahertz metamaterial absorbers (TMMAs) have been devised for applications in communication, security, imaging, and sensing [9,10,11,12,13]. Particularly, TMMAs are increasingly recognized for their potential as highly-sensitive sensors. However, achieving heightened sensitivities and lowered detection limits often necessitates a high quality factor (Q factor) in the absorber [14]. At present, there are two main methods to improve the Q factor. One effective strategy involves the cascading of multiple layers, but it usually introduces complexity into the design, increases costs, and limits applicability [15]. Another approach involves employing asymmetric structures to induce Fano-type resonance [16]. However, these asymmetric structures operate only under specific polarizations. Hence, the way to obtain an easily fabricated, polarization-insensitive TMMA with an ultrahigh Q factor is still lacking.

Graphene, as a typical representative of 2D materials, has significant contributions to improving the Q factor and detection sensitivity of TMMA sensors, owing to its outstanding characteristics [17,18,19,20]. The utilization of graphene in TMMA facilitates the efficient absorption of THz wave energy [21,22,23], attributed to the high confinements of the electromagnetic resonance excited in graphene [24]. In contrast to noble metals, graphene exhibits heightened sensitivity to environmental changes with lower loss [25]. It has been demonstrated in our previous work that the use of graphene can effectively enhance the Q factor of TMMA. The designed graphene-enhanced TMMA exhibited a maximum Q factor of 133.12, accompanied by a corresponding figure of merit (FoM) reaching 18.28 per refractive index unit (RIU^−1^) [26]. Furthermore, adjusting the conductivity of graphene is achievable by applying a biased gate voltage to modify its Fermi level. This property enables graphene-based TMMAs to attain dynamic tunability [27,28].

Recently, various types of graphene-based TMMA have been proposed. In 2020, Barzegar-Parizi et al. designed a multi-band plasmonic absorber by employing a patterned graphene as its metamaterial resonator. When used for sensing, the proposed structure exhibited a theoretical sensitivity of 0.11 THz/RIU while attaining a Q factor of 50 and a maximum figure of merit (FoM) of 5.3 RIU^−1^ [29]. In 2020, Rezagholizadeh et al. introduced a THz refractive index sensor using periodic arrays of graphene disks. It exhibited a numerically simulated sensitivity of 1.57 THz/RIU, coupled with a Q factor of 80 and a maximum FoM of 24.5 RIU^−1^ [30]. In 2023, Ma et al. proposed a tunable graphene TMMA as a bifunctional sensor. This sensor demonstrated a theoretical frequency sensitivity of 1.43 THz/RIU, accompanied by a maximum Q factor of 57.53 and an FoM of 9.65 RIU^−1^ [31]. In the same year, Ma et al. proposed a highly sensitive terahertz sensor based on graphene layers featuring a circular air hole in the center. Simulation results revealed maximum sensitivity, Q factor, and FoM values of 2.372 THz/RIU, 179.95, and 64.62 RIU^−1^, respectively [32]. Note that the TMMAs mentioned above all rely on graphene patterning. However, achieving precise graphene patterning is quite difficult, especially for large areas. Jia et al. designed an actively tunable graphene-based metal–insulator–metal (MIM) TMMA, utilizing a continuous graphene layer to eliminate the graphene patterning process. However, the fields are mostly constrained in the metal, leading to a limited Q value of 32 and an FoM of 33 RIU^−1^ [33].

Here, a TMMA consisting of a continuous, pattern-free graphene with ultrahigh, polarization-independent Q factors reaching above 1700 is demonstrated. The graphene sheet is overlaid on an Al metal array, forming a two-dimensional graphene layer that supports strong localized surface plasmon polaritons (LSPPs) whose fields are tightly confined in the graphene, minimizing loss. Polarization independency is achieved with structural rotational symmetry. The simulation also highlights the capacity of the TMMA for refractive index sensing, with an outstanding FoM of 365.85 RIU^−1^. A dynamic tunability of the resonance frequency from ~2.25 to ~3.25 THz is also achievable with the change *E_f_* of graphene. The presented TMMA shows great potential in biological, chemical, and gas sensing.

## 2. Design of the TMMA

**Figure 1:**
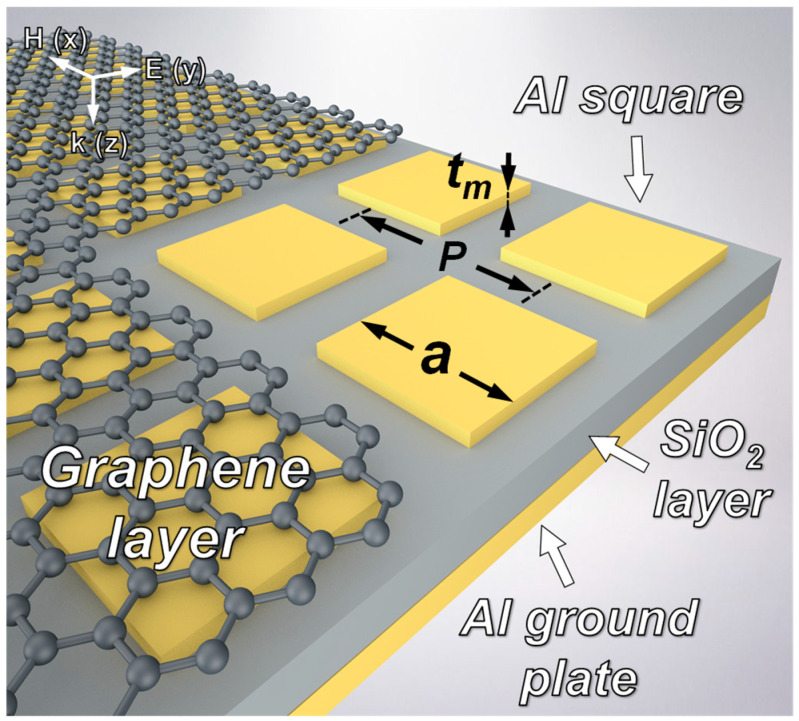
Structure schematic of the proposed graphene-based TMMA. Structural parameters: *P* = 65 μm, *a* = 40 μm, and *t_m_* = 0.1 μm.

A schematic representation of the proposed graphene-based TMMA is shown in Figure 1. The absorber is based on a graphene-covered MIM metamaterial structure with monolayer graphene, an array of cubic Al resonators, a SiO_2_ dielectric layer, and an Al ground plane. The refractive index of the SiO_2_ dielectric layer is 2.0 + 0.025*i* [34]. Geometric parameters are defined as follows: *P* = 65 μm, *a* = 40 μm, *t_m_* = 0.1 μm, *t_d_* = 4.2 μm, and *t_g_* = 0.2 μm. Here, ‘*P*’ represents the unit cell periodicity, ‘*a*’ is the length of a cubic Al resonator, ‘*t_m_*’ indicates the thickness of the cubic Al resonators, and ‘*t_d_*’ denotes the thickness of the SiO_2_ dielectric layer. The bottom ground plane employs an Al plate with a thickness of *t_g_* = 0.2 µm, serving as a reflecting mirror. This ensures the comprehensive reflection of the impinging terahertz wave, effectively suppressing its transmission. The proposed graphene-based TMMA can be conveniently manufactured utilizing advanced micro- and nanofabrication processes. At first, a layer of Al is deposited onto a Si wafer to function as the ground plane. Next, the Al ground plane is covered with a SiO_2_ dielectric layer through plasma-enhanced chemical vapor deposition. Subsequently, lithography and electron beam evaporation techniques are employed to create the array of cubic Al resonators. Finally, monolayer graphene is transferred onto the Al resonators through a wet transfer method to form the whole structure [35].

In the THz range, the conductivity of graphene (*σ_g_*) is composed of the intra-band and inter-band contributions [36]:

(1) $$\sigma_{g}=\sigma_{intra}+\sigma_{inter}$$

(2) $$\sigma_{intra}=\frac{ie^{2}k_{B}T}{\pi \hbar^{2}\left(\omega +i\tau^{-1}\right)}\left[\frac{E_{f}}{k_{B}T}+2ln(e^{-\frac{E_{f}}{k_{B}T}}+1)\right]$$

(3) $$\sigma_{inter}=\frac{ie^{2}}{4\pi \hbar^{2}}ln\left[\frac{2\left|E_{f}\right|-\hbar (\omega +i\tau^{-1})}{2\left|E_{f}\right|+\hbar (\omega +i\tau^{-1})}\right]$$

where *i* is the imaginary unit, *e* is the elementary charge, *k_B_* refers to the Boltzmann constant, and *T* is the ambient temperature. ℏ refers to the approximate Planck’s constant, *ω is* denoted as the angular frequency of the incident light, *E_f_* represents the Fermi level of the graphene, and *τ* is the carrier relaxation time of graphene. Previous studies have indicated that at bands at low optical frequencies, e.g., THz, the *E_f_* of graphene is much larger than *ℏω*, resulting in the negligible contribution of *σ_inter_*. Therefore, the conductivity of graphene can be simplified as follows [37]:

(4) $$\sigma_{g}=\sigma_{intra}=\frac{e^{2}\left|E_{f}\right|}{\pi \hbar^{2}}\frac{i}{\left(\omega +i\tau^{-1}\right)}$$

In this study, the initial physical parameters of graphene are *E_f_* = 0.5 eV, *τ* = 1.0 ps, and *t_Gr_* = 0.34 nm [38,39].

## 3. Results and Discussions

The finite element method is employed using COMSOL Multiphysics version 5.5 to simulate the absorption and sensing characteristics of the proposed graphene-based TMMA. Periodic boundary conditions are used to simulate periodicity. The excitation plane wave upon the TMMA is at normal incidence. In order to eliminate non-physical reflections, A perfectly matched layer (PML) is employed in the Z-direction. The mesh size is adjusted to meet convergence and accuracy requirements. According to the principle of energy conservation, the absorption rate *A* can be calculated as follows [40]:

(5) A=1−R−T

where *R* represents the reflectance rate. *T* represents the transmittance rate. As the bottom Al ground plane entirely impedes the transmission of THz waves (*T* = 0), the overall absorption is computed as *A* = 1 − *R*. Another key metric for absorber assessment is the Q factor, expressed as follows [41]:

(6) $$Q=\frac{f}{FWHM}$$

where *FWHM* is the full width at half maximum of the resonant absorption peak. The *f* is the resonant frequency of the absorption peak. In general, a metamaterial absorber with a large Q value indicates considerable potential for applications in the sensing field [42].

### 3.1. Analyses of Resonance Modes

**Figure 2:**
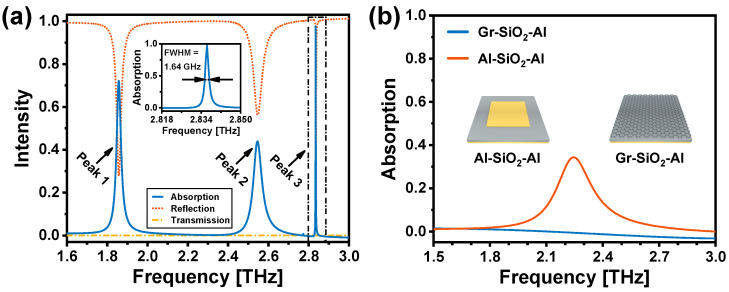
(**a**) Simulated absorption, reflection, and transmission spectra of the graphene-based TMMA. The inset is the zoom-in plot of the Peak 3, showing a narrow FWHM of 1.64 GHz. (**b**) Simulated absorption spectrum of the Al-SiO_2_-Al (orange line) and graphene-SiO_2_-Al (blue line) structures.

The numerically simulated absorption spectrum of the graphene-based TMMA is depicted in Figure 2a. There are three absorption peaks at 1.86 THz (Peak 1), 2.55 THz (Peak 2), and 2.84 THz (Peak 3). Notably, Peak 3 exhibits the highest absorption rate of 97.71%. The inset provides a magnified view near Peak 3, revealing that it has a narrow FWHM of 1.64 GHz. The corresponding Q factor of Peak 3 is 1730, surpassing that of Peak 1 and Peak 2 by more than 25 and 35 times, respectively. Additionally, for purposes of comparison, the absorption spectra of Al-SiO_2_-Al and graphene-SiO_2_-Al metamaterial structures are investigated under identical conditions, as depicted in Figure 2b. The Al-SiO_2_-Al metamaterial structure exhibits a single absorption peak at 2.24 THz, with a relatively low absorption rate of 34.20%. Its FWHM is calculated to be 228.46 GHz, corresponding to a low Q factor of 10. Conversely, the graphene-SiO_2_-Al structure does not exhibit a resonance absorption peak.

**Figure 3:**
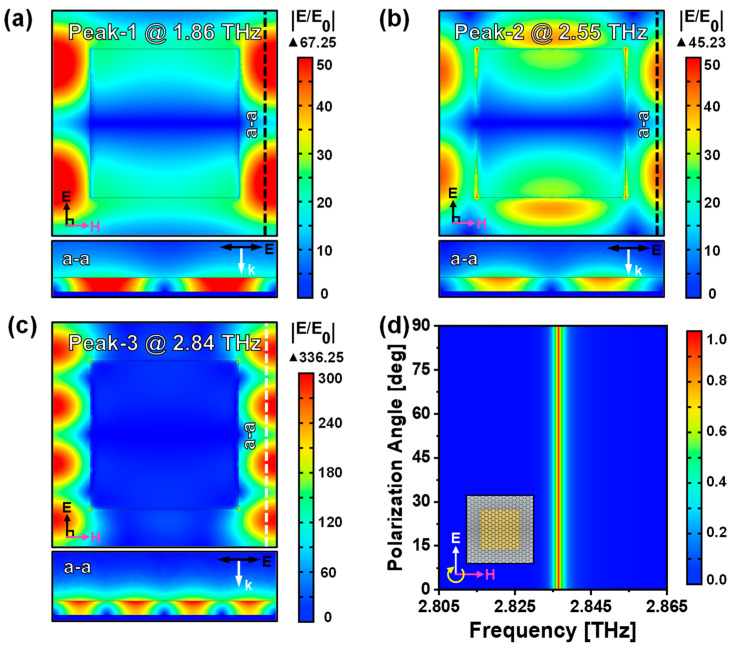
(**a**–**c**) Plotted |E/E_0_| field distributions of the graphene-based TMMA at (**a**) 1.86 THz (Peak 1), (**b**) 2.55 THz (Peak 2), and (**c**) 2.84 THz (Peak 3). Upper part is the top view of the electrical field distribution at the surface of graphene; lower part is the cross-sectional view of the electrical field distribution, extracted along line a-a in the upper part. (**d**) Simulated absorption spectra with varying polarization angles of electrical fields from 0° to 90°.

To attain a more profound understanding of the absorption mechanism for the proposed graphene-based TMMA, Figure 3a–c plot the |E/E_0_| electrical field enhancement distribution for Peak 1, 2, and 3, respectively. The electrical fields are concentrated near the graphene for all three modes. Additionally, for Peak 1 and 2, part of the fields is constrained in the Al resonators, indicating more loss, which accounts for their much lower Q factors compared with that of Peak 3. Moreover, the interference patterns in the graphene are expected to be caused by the interference of the LSPPs. Their different periodicity corresponds to different resonance frequencies. Note that for Peak 3, although most of the fields are concentrated near the graphene, the Al array is essential for exciting the resonance, as is shown by Figure 2b that no resonance is excited when the Al resonators are absent. The primary function of the Al array is to adjust the plane wave vector component through interaction with incident light. This adjustment facilitates the achievement of wave vector matching within the two-dimensional graphene metasurface, leading to the excitation of strong LSPPs. Hence, ultrahigh Q factors are achieved without the need for graphene patterning. Furthermore, for Peak 3, due to the rotational symmetry of the mode, the absorption properties exhibit insensitivity to the polarization angles, as is illustrated in Figure 3d. Owing to the excellent properties of the resonance mode at Peak 3, subsequent investigations in this study are predominantly concentrated on Peak 3.

### 3.2. Tunability of the Graphene-Based TMMA

As is known, the absorption capabilities of TMMAs are significantly influenced by their structural parameters [43,44]. Here, the impact of the dielectric layer thickness (*t_d_*), unit cell period (*P*), and side length of the metal resonator (*a*) on the performances of the graphene-based TMMA is systematically investigated.

**Figure 4:**
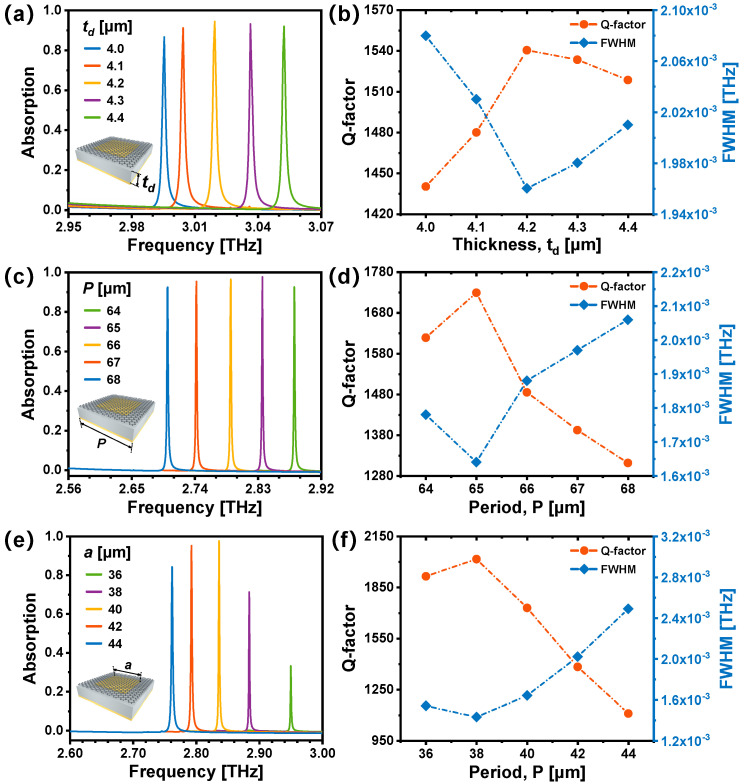
Simulated absorption spectra of the graphene-based TMMA with different (**a**) SiO_2_ dielectric thickness *t_d_*, (**c**) period *P*, and (**e**) side length of the cubic Al resonators *a*. (**b**,**d**,**f**) calculated FWHMs and Q factors corresponding to (**a**), (**b**), and (**c**), respectively.

Figure 4a illustrates the numerically simulated results on the absorption properties of the graphene-based TMMA versus the thickness of the SiO_2_ dielectric layer (*t_d_*). The absorption efficiency maintains high as *t_d_* increases, accompanied by a blue shift of the resonant frequency due to changes in the overall effective refractive index of the absorber [45,46]. Figure 4b displays the simulated FWHMs and Q factors corresponding to different *t_d_*. When the dielectric thickness is 4.2 μm, the FWHM reaches its minimum at 1.96 GHz, while the Q factor attains its maximum at 1540. Similarly, the impact of varying the period *P* of the graphene-based TMMA is explored (*t_d_* = 4.2 μm). Simulation results are illustrated in Figure 4c,d. When period *P* increases from 64 to 68 μm, a red shift happens due to geometric scaling. When the period *P* reaches 65 μm, the minimum FWHM and maximum Q factor is 1.64 GHz and 1730, respectively. Figure 4e,f exhibit the variation in absorption characteristics as the side length of the cubic Al resonator (*a*) is increased from 36 to 44 μm (*t_d_* = 4.2 μm, *P =* 65 μm). A red shift of the resonance frequency is observed, and the absorption rate of the resonance peak changes notably. Despite the FWHM decreasing to 1.43 GHz and the Q value increasing to 2017 at a side length of 38 μm, the absorption rate markedly drops to 71.23%. Thus, the optimal structural parameters are determined as follows: *t_d_* = 4.2 μm, *P* = 65 μm, and *a* = 40 μm.

**Figure 5:**
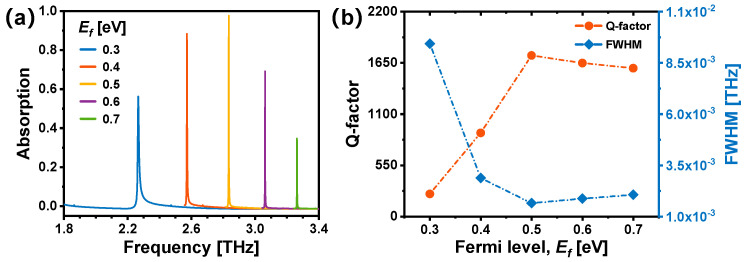
(**a**) Simulated absorption spectra of the graphene-based TMMA with different Fermi levels *E_f_* of graphene. (**b**) Calculated Q factors and FWHMs corresponding to the modes in (**a**) with different *E_f_*.

Additionally, the absorption properties of the graphene-based TMMA are notably affected by the Fermi level *E_f_* of graphene, which can be adjusted via chemical doping or electrical gating [19,25]. As is shown in Figure 5a, when *E_f_* increases from 0.3 to 0.7 eV, a blue shift from ~2.25 to ~3.25 THz is observed in the resonance peak, accompanied by significant variations in absorption rates. Figure 5b shows that when *E_f_* = 0.5 eV, the FWHM attains its minimum value of 1.64 GHz, corresponding to the highest Q value of 1730. The adjustment of the Fermi level *E_f_* inherently implies the modulation of graphene’s carrier concentration. Through electronic injection or depletion, the changes in carrier concentration can further influence the complex refractive index of graphene, thereby inducing alterations in its absorption characteristics [47]. This opens the possibility of the dynamic tuning of the TMMA.

### 3.3. Sensing Capabilities of the Graphene-Based TMMA

**Figure 6:**
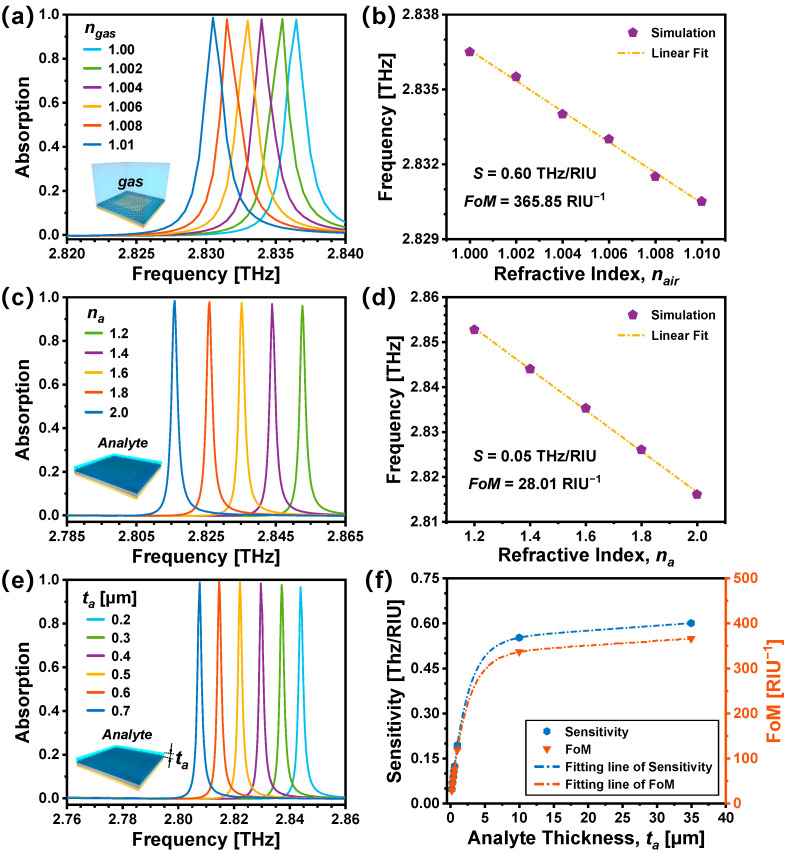
(**a**) Simulated absorption spectra of the graphene-based TMMA in environments with different refractive indexes *n_gas_*. (**b**) Resonance frequency versus *n_gas_*. (**c**) Absorption spectra obtained with an overlayer of analytes with different refractive indexes *n_a_*. (**d**) Resonance frequency versus *n_a_*. (**e**) Simulated absorption spectra with overlayers of different thicknesses *t_a_* (*n_a_* = 1.4). (**f**) Sensitivity and FoM versus *t_a_*.

Here, simulations are conducted to investigate the sensing applicability of the proposed graphene-based TMMA. The gas sensing characteristics of the proposed graphene-based TMMA are initially explored by adjusting the refractive indices (*n_gas_*) of the surrounding environment. Typical gases demonstrate refractive indices ranging from 1.00 to 1.01 [48,49]. Therefore, *n_gas_* is varied from 1.00 to 1.01, with intervals of 0.002. Figure 6a illustrates, as *n_gas_* increases from 1.00 to 1.01, the resonance absorption peak experiences a red shift, while the absorption rate remains nearly constant. To quantify this absorption peak shift, the frequency sensitivity (S), as a crucial index for assessing sensing capabilities, is calculated as S=∆f/∆n. Here, Δ*f* and Δ*n* represent the variation in the resonant frequency and refractive index, respectively. Another crucial index used to estimate the sensing performance is the FoM, which is expressed as FoM=S/FWHM. As illustrated in Figure 6b, for sensing gas, the sensitivity S is 0.60 THz/RIU, and the FoM could reach as high as 365.85 RIU^−1^.

Subsequently, the potential for the graphene-based TMMA to serve as liquid- or bio-sensors is explored. Prior studies indicate that refractive indices of liquids, biomolecules, and cells typically range from 1.3 to 2.0 [36,41,47,50]. Assuming an overlayer thickness *t_a_* of 0.2 μm, the refractive indices *n_a_* of the overlayer analytes are adjusted within the range of 1.2 to 2.0 at an interval of 0.2. The absorption spectra are numerically simulated, as shown in Figure 6c. It can be observed that the resonance peak exhibits a red shift with increased *n_a_*. Figure 6d shows that its frequency sensitivity and FoM are 0.05 THz/RIU and 28.01 RIU^−1^, respectively. Compared with gas sensing, the frequency sensitivity S and FoM exhibit relatively smaller values, which is attributed to the finite thickness of the analyte layer. The finite analyte volume results in a weaker light–matter interaction. To further explore this phenomenon, the frequency sensitivity S and FoM are calculated for different thickness analyte layers. The results are shown in Figure 6e,f. The resonance peak also exhibits a red shift with increased *t_a_.* As demonstrated in Figure 6f, the implementation of small values for analyte thickness leads to a swift degradation in sensitivity S and the FoM, whereas an increase in analyte thickness displays a tendency toward saturation.

Table 1 compares the previously proposed graphene-based sensors with the presented TMMA sensor [29,30,31,32,51,52,53]. It can be observed from the table that the Q value and FoM of the graphene-based TMMA sensor is substantially higher due to field confinement in the graphene. Meanwhile, the polarization insensitivity of this sensor facilitates its practical application. Consequently, the presented graphene-based TMMA demonstrates outstanding sensing performance, offering promising prospects in future sensing applications. 

## 4. Conclusions

In conclusion, a tunable TMMA with ultrahigh Q factors consisting of a continuous, pattern-free graphene sheet is proposed and investigated. The monolayer graphene is covered on an Al cube array, without the graphene patterning, which is beneficial for simplifying the preparation process. The structure supports strong LSPPs highly confined in the graphene, minimizing loss. Additionally, its resonance modes of absorption peaks can be flexibly adjusted via modifications in geometric parameters or by tuning the *E_f_* of graphene. Theoretical results indicate that the graphene-based TMMA exhibits a maximum Q factor of 1730, a frequency sensitivity of 2.84 THz/RIU, and an FoM of 365.85 RIU^−1^. Due to rotational symmetry, its absorption properties are insensitive to the polarization angles. The presented graphene-based TMMA exhibits superior sensing properties and versatility for both gas and liquid sensing, offering promising prospects in future sensing applications.

## Figures and Tables

**Table 1:** Absorption performance comparisons of different graphene-based TMMA reported in previous works.

Structure	Year	Frequency (THz)	FoM (RIU^−1^)	Q Factor	Ref.
Monolayer graphene ring	2021	5.55	8.75	27.75	[51]
Graphene and Au SRR	2021	2.5	9.48	49.2	[52]
Graphene disks	2020	5.9	5.3	50	[29]
Circular graphene disks	2015	9.01	6.57	59	[53]
Graphene disks	2020	6	24.5	80	[30]
Graphene and InSb cylinder	2023	8.53	9.65	59.53	[31]
Graphene layer with circular holes	2023	2.372	64.62	179.95	[32]
Al resonators with unpatterned graphene		2.84	365.85	1730	This work

## Data Availability

The data presented in this study are available on request from the corresponding author.
